# Association of abnormal high-density lipoprotein cholesterol with depressive symptoms and variations by gender: Preliminary results from Singapore’s PREDICT study

**DOI:** 10.1192/j.eurpsy.2026.10583

**Published:** 2026-07-31

**Authors:** J. A. Vaingankar, B. Tan, N. Chandwani, P. Satghare, Y. J. Zhang, A. Jeyagurnathan, E. Abdin, E. Samari, S. Gunasekaran, S. Archana, M. Ramu, S. Shahwan, R. Sambasivam, Y. M. Mok, S. A. Chong, M. Subramaniam

**Affiliations:** 1 Research; 2 Department of Mood and Anxiety, Institute of Mental Health, Singapore, Singapore

## Abstract

**Introduction:**

Substantial evidence confirms a linear relationship between low-density lipoprotein and depression. However, findings on the association of abnormal high-density lipoprotein cholesterol (HDLc) with depression are inconclusive, with both low and high levels of HDLc implicated in severe symptoms and suicidality. Additionally, gender differences in this association remain underexplored.

**Objectives:**

This study evaluated the association between abnormal serum HDLc and depressive symptom severity and investigated whether the association varied by gender in a sample of multi-ethnic adults in Singapore.

**Methods:**

Descriptive and multivariable analyses were conducted on data collected from participants of a longitudinal cohort - The PREDICT study. Participants were aged 18-75 years, of Chinese, Malay, or Indian ethnicity, with major depressive disorder (MDD) or general population without any psychiatric diagnosis. The primary outcome was depressive symptom score assessed with the Patient Health Questionnaire (PHQ)-9. Its association with abnormal serum HDLc levels (categorised into ordinal variable for abnormal low (AL), normal (N), and abnormal high (AH) HDL) and gender differences were investigated after adjusting for age, race, and known confounders of lipid abnormality (body mass index, mental health treatment, and history of chronic conditions).

**Results:**

The study sample comprised 212 participants with a mean age of 38.1 (SD 13) years. In all, 59.4% were women, 76.9% were Chinese, and 51.4% were diagnosed with MDD. Total PHQ-9 score ranged from 1- 34, with a mean score of 7.8 (SD 7); 19.8% met criteria for moderately severe to severe depression. The prevalence of abnormal HDLc was 58% with AL-HDLc and AH-HDLc observed in 11.8% and 46.2% of the sample, respectively. Compared to persons with N-HDLc, AL-HDLc was significantly and directly associated with symptom severity (β = 4.4, 95% CI:1.2 to 7.7, p = 0.008), while an inverse association was observed with AH-HDLc (β = −2.6, 95% CI:‒4.7 to −0.5, p = 0.015). The significant direct association between AL-HDLc and symptom severity was observed only among men, whereas AH-HDLc showed a significant inverse relationship only in women. The association between AL-HDLc remained significant after adjusting for confounders in the overall sample and among men.

**Conclusions:**

Study results indicate that low HDLc is linked to more severe depressive symptoms, whereas high levels of HDLc have an inverse relationship. The latter contrasts with emerging literature on adverse outcomes of depression associated with high HDLc. Gender differences exist in these associations, which need to be investigated further and replicated in a larger sample. Nevertheless, these preliminary findings underscore the importance of maintaining healthy HDLc levels, which may lower morbidity in depressive disorders.

**Disclosure of Interest:**

None Declared

